# Correction: Maachia, L.; *et al.* Assessment of the Petrochemical Industry Pollution on Skikda Bay, Algeria. *Int. J. Environ. Res. Public Health* 2005, *2*, 463–468

**DOI:** 10.3390/ijerph120505483

**Published:** 2015-05-21

**Authors:** Leila Maachia, Nafissa Boutefnouchet, Noureddine Bouzerna, Houria Chettibi

**Affiliations:** 1 Faculté des Sciences, Département de biologie, Université 20 Août 1955, Skikda, B.P. 26 route d’El-Hadaiek, 21000, Algeria; 2 Faculté des Sciences, Département de Biochimie, Université Badji Mokhtar, B.P. 12, Sidi-Amar, Annaba 23200, Algeria; E-Mail: chettibih195@yahoo.com

We wish to make the following changes to the published article [1], agreed upon by all authors: Leila Maachia has been added as co-author. The corrected author list should therefore read: Leila Maachia, Nafissa Boutefnouchet, Noureddine Bouzerna and Houria Chettibi. Author contributions are corrected accordingly:
